# Elimination of mother-to-child transmission of HIV and Syphilis (EMTCT): Process, progress, and program integration

**DOI:** 10.1371/journal.pmed.1002329

**Published:** 2017-06-27

**Authors:** Melanie Taylor, Lori Newman, Naoko Ishikawa, Maura Laverty, Chika Hayashi, Massimo Ghidinelli, Razia Pendse, Lali Khotenashvili, Shaffiq Essajee

**Affiliations:** 1 Department of Reproductive Health and Research, World Health Organization, Geneva, Switzerland; 2 Division of STD Prevention, Centers for Disease Control and Prevention, Atlanta, Georgia, United States; 3 United States Centers for Disease Control and Prevention, Phnom Penh, Cambodia; 4 Division of Communicable Diseases, World Health Organization Regional Office for the Western Pacific, Manila, Philippines; 5 HIV Department, World Health Organization, Geneva, Switzerland; 6 Department of Communicable Diseases and Health Analysis, Pan American Health Organization, Washington DC, United States; 7 Regional Office for South-East Asia, World Health Organization, Delhi, India; 8 European Regional Office, World Health Organization, Copenhagen, Denmark

## Abstract

Melanie Taylor and colleagues discuss progress towards eliminating vertical transmission of HIV and syphilis.

**Summary points**

- The dual elimination of mother-to-child transmission (EMTCT) of HIV and syphilis has been identified as a global public health priority.
- Impact criteria required by WHO for validation of EMTCT include: for HIV, ≤50 new pediatric infections per 100,000 live births and a transmission rate of either <5% in breastfeeding populations or <2% in nonbreastfeeding populations; and for syphilis, ≤50 cases of congenital syphilis per 100,000 live births.
- Required process criteria for validation of EMTCT include: 95% of pregnant women to receive antenatal care (ANC); 95% of pregnant women to receive HIV and syphilis testing in pregnancy; and 95% of pregnant women diagnosed with HIV or syphilis to receive treatment.
- WHO requires that HIV and syphilis EMTCT processes and indicators are achieved within a context of human rights, gender equality, and community engagement.
- Among countries validated for EMTCT, elimination activities including case reporting, surveillance, and laboratory quality assurance for congenital syphilis were less developed than those for HIV.
- Countries with high and low maternal HIV and syphilis prevalence face different challenges related to achieving the impact and process indicator targets of EMTCT.
- Innovative approaches such as use of rapid dual HIV/syphilis point-of-care testing in antenatal care can improve maternal screening coverage, particularly for syphilis.
- Commitment to the triple EMTCT of HIV, syphilis, and hepatitis B is forthcoming from multiple regions and WHO.

## Introduction

Nearly 1 million syphilis infections occur among pregnant women globally each year. These syphilis-affected pregnancies resulted in an estimated 350,000 adverse birth outcomes due to congenital syphilis in 2012 [1]. More than 1.4 million pregnant women are infected with HIV, and mother-to-child transmission (MTCT) of HIV is estimated to have resulted in over 150,000 infant cases in 2015 [2]. Untreated maternal syphilis results in congenital syphilis in over half of affected pregnancies and can lead to early fetal loss, premature birth, stillbirth, low birth weight, complications from infection, and neonatal death [3]. Over half of infants vertically infected with HIV die before the age of 2 years [2]. Antenatal screening for syphilis and HIV, and treatment for pregnant women infected, prevents MTCT and aligns with the Sustainable Development Goal (SDG) targets of ending preventable deaths of newborns and children under 5 years of age, ensuring universal access to sexual and reproductive healthcare services, and achieving universal health coverage (UHC) [4] as well as the Joint United Nations Start Free-Stay Free-AIDS Free initiative [5]. In May 2016, the World Health Assembly endorsed 3 new global health strategies (2016–2021) on HIV, sexually transmitted infections (STIs), and hepatitis. These strategies call for member states and WHO to work together towards goals of 0 new HIV infections in infants by 2020, the elimination of congenital syphilis as a public health threat by 2030, and achieving a 0.1% prevalence of hepatitis B surface antigen (HBsAg) among children by 2030 [6–8].

The global health community, led by WHO, has identified dual elimination of mother-to-child transmission (EMTCT) of HIV and syphilis as a priority. In 2007, WHO launched an initiative for the elimination of congenital syphilis [9] and, in 2011, the agency set a target to reduce MTCT of HIV by 90% [10]. In 2014, WHO developed global guidance containing integrated processes and criteria for validation of EMTCT of HIV and syphilis [11], and 1 year later Cuba became the first country to be validated for having achieved EMTCT of HIV and syphilis [12]. Other countries have followed and in 2016, Thailand and Belarus were validated for EMTCT of HIV and syphilis, Moldova was validated for EMTCT of syphilis, and Armenia was validated for EMTCT of HIV [13]. Numerous countries have EMTCT plans in place [14,15], and others have committed to applying for EMTCT validation by a target date [16]. The 5 countries that have been validated for achievement of EMTCT have similar strategies in place to ensure universal and equitable antenatal care (ANC) services that include HIV and syphilis testing and treatment at no cost to pregnant women [17]. In this respect, the achievement of EMTCT among the validated countries reflects progress towards UHC [18,19]. The essential dimensions of UHC—population coverage (who is covered?), service coverage (what services are covered?), financial coverage (what costs are covered?), and protection and quality of services—are key components of the validation of EMTCT, which are reviewed in detail during in-country assessments [19]. Herein, the EMTCT validation process and lessons learned from validation of the aforementioned countries are described.

## EMTCT process and indicators

**Fig 1:**
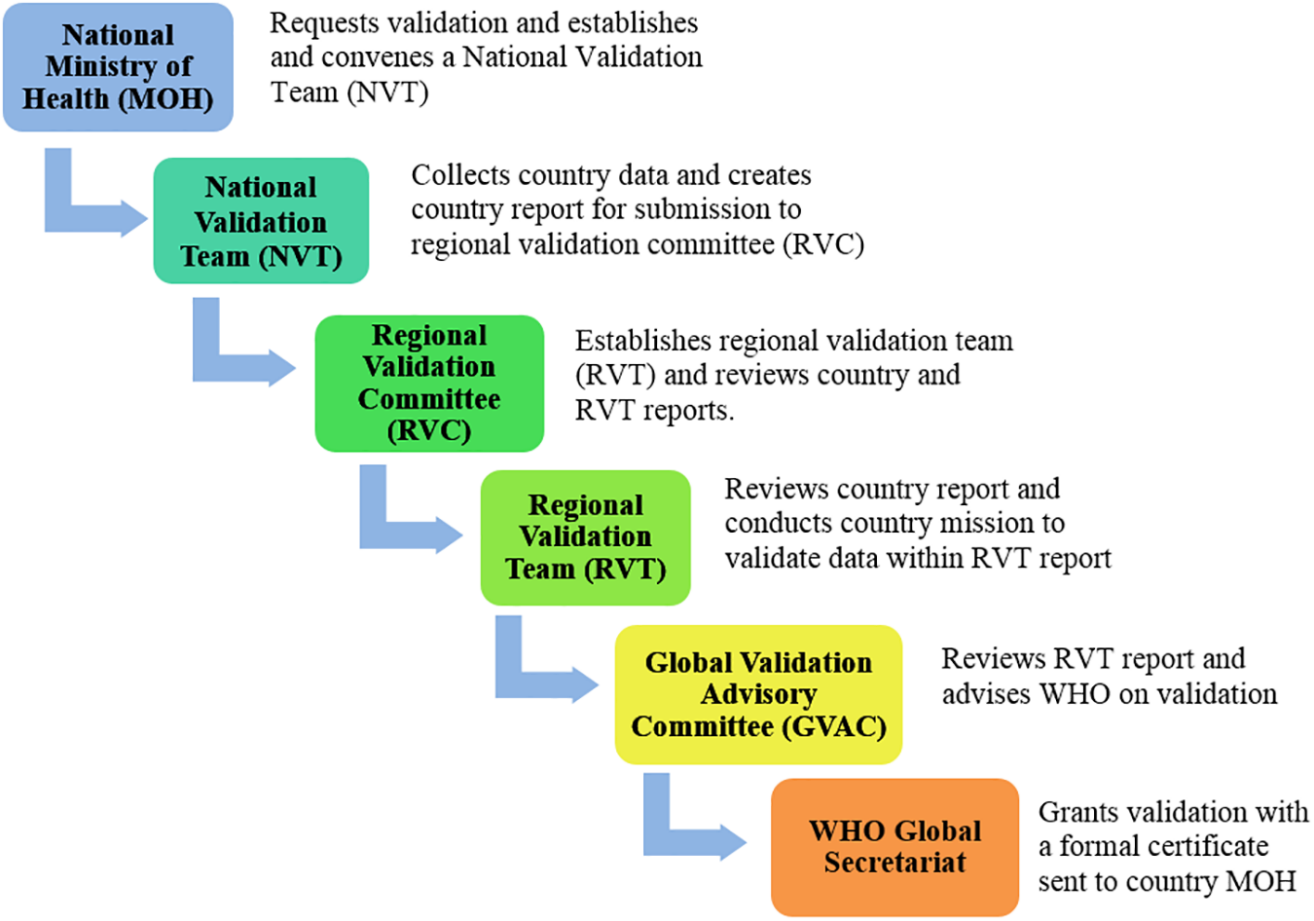
Country review process for elimination of mother-to-child transmission.

WHO, in collaboration with the Joint United Nations Programme on HIV/AIDS (UNAIDS), UNICEF, and the United Nations Population Fund (UNFPA), has developed standardized processes and criteria to validate EMTCT of HIV and syphilis that emphasize country-led accountability, analytic and programmatic rigour, and multilevel collaboration [11]. This global guidance outlines the process, indicator targets, and available support for programmatic advancement towards achieving the benchmarks required for validation. The validation process consists of a series of national-, regional-, and global-level program and data reviews (Fig 1, S1 Fig). Regional and global validation committee secretariat functions are performed by WHO Regional Offices and Headquarters, in partnership with UNAIDS, UNFPA, and UNICEF. Subsequently, WHO headquarters monitors maintenance of EMTCT of HIV and syphilis annually through routine global reporting mechanisms already in place and with additional reports from validated countries.

## Criteria for EMTCT validation

WHO has established global EMTCT impact and service delivery (process) targets (Box 1). Impact targets must be met for at least 1 year prior to EMTCT validation assessment. Coverage of antiretroviral treatment in HIV-infected pregnant women is set at 90% in current EMTCT validation guidance. This criterion for maternal HIV treatment coverage will be increased from ≥90% to ≥95% in the forthcoming revised EMTCT validation guidance expected later in 2017. All of the countries that have achieved validation thus far reached the ≥95% target. Process targets must have been achieved for the 2 consecutive years prior to validation. Countries must ensure that validation criteria have been met in a manner consistent with human rights [20].

**Box 1. EMTCT impact and process targets [10]**

The minimum EMTCT impact targets are:

1. For HIV, ≤50 new pediatric infections per 100,000 live births and a transmission rate of either <5% in breastfeeding populations or <2% in nonbreastfeeding populations;
2. For syphilis, ≤50 cases of congenital syphilis per 100,000 live births.

Specific levels of service delivery also need to be met to accomplish EMTCT of HIV and syphilis.

There are 4 process targets:

1. ANC coverage (at least 1 visit) of ≥ 95%
2. Coverage of HIV and/or syphilis testing of pregnant women of ≥ 95%
3. Antiretroviral treatment coverage of HIV-positive pregnant women of ≥ 95%*
4. Treatment of syphilis-seropositive pregnant women of ≥ 95%.

*Antiretroviral treatment coverage of pregnant women will increase from 90% to 95% with pending revision of the global guidance on EMTCT.

## Lessons learned from country validation

During the validation process, a country must demonstrate that all pregnant women and infants exposed to HIV or syphilis have universal access to essential interventions to prevent MTCT (population coverage and service coverage). Quality of interventions including laboratory services is also assessed. Financial barriers, out-of-pocket payment, and financial support for vulnerable populations are evaluated to ensure equitable access (S2 Fig). These criteria must be sufficiently fulfilled for the country to be validated for EMTCT. A selection of EMTCT program attributes from the 5 validated countries is presented in Table 1. Validation teams working within countries identified challenges encountered by countries within the 4 EMTCT areas of program services, data and surveillance, laboratory services, and human rights and community engagement. Some of these are highlighted in this paper. Overall, syphilis surveillance and laboratory services were less developed than those of HIV. Additional challenges encountered when delivering EMTCT services among special populations are described.

**Table 1:** Program and country attributes from 5 EMTCT validated countries.

Country	Date	Program and country attributes towards EMTCT
	(Type of Validation)	
Cuba	June 2015 (HIV & Syphilis)	1. Free testing for HIV and syphilis at multiple time points during pregnancy for women and their partners
		2. High per capita ratio of clinical care providers
		3. Minimal health disparities
		4. Essentially no migrant populations
		5. Efforts to prevent congenital syphilis began in 1970s
		6. Routine antenatal HIV testing started in 1987
Thailand	May 2016 (HIV & Syphilis)	1. Near universal healthcare access
		a. 99.9% of Thai citizens have government supported insurance
		b. Additional 1.4 million non-Thai migrants have health insurance
		2. All PMTCT services (ANC, screening and treatment for HIV and syphilis) financed through national budget with free access for migrants
		3. Routine antenatal screening for syphilis for more than 30 years
		4. Routine antenatal screening for HIV for approximately 20 years
		5. The only country thus far with a generalized epidemic to achieve EMTCT
Belarus	June 2016 (HIV & Syphilis)	1. Universal free access to ANC
		2. Free HIV and syphilis testing for all women (including prisoners)
		3. Free ART and syphilis treatment for pregnant women and newborns (including asylum seekers, migrants, applicants for residence, and refugees from conflict zones)
		4. Provides formula free of charge to all infants born to HIV-positive mothers
		5. Free emergency care services for pregnant women, including noncitizens
Armenia	June 2016 (HIV only)	1. All citizens have access to free medical care and social assistance
		2. Noncitizens have access to free emergency services
		3. ANC, HIV testing and treatment, and delivery service free to pregnant women (citizens and noncitizens)
		4. Activities to involve male partners in testing and treatment
		5. Achieved EMTCT despite 70% of HIV cases reported in 2011–2015 being associated with migrants and their partners
Moldova	June 2016 (syphilis only)	1. Universal access to basic health services
		2. Well-developed network of primary care facilities
		3. Integrated national program for STIs including HIV
		4. ANC services, testing and treatment of syphilis available at no cost to pregnant women and infants
		5. Amendments to legal frameworks to improve laws on domestic violence, sexual and reproductive rights, and patient rights ensuring equitable delivery of EMTCT services

**Abbreviations:** ANC, antenatal care; ART, antiretroviral therapy; EMTCT, elimination of mother-to-child transmission; PMTCT, prevention of mother-to-child transmission; STI, sexually transmitted infection.

### Program services

Ensuring maternal screening, treatment, and infant follow-up are cornerstones of EMTCT programming. Variations in infant follow-up processes and duration were encountered among countries applying for validation. Countries with very low HIV prevalence faced challenges in documenting infant diagnosis and infant feeding practices, especially in key at-risk populations. In countries where breastfeeding or mixed feeding is the norm, infants should be followed for up to 18 months for establishment of final HIV negative status, while in formula-fed populations a negative virological test at 3 months is considered definitive [21]. Identification and surveillance monitoring systems for congenital syphilis cases (particularly stillbirths) were less robust than those for HIV, and case definitions for congenital syphilis varied among countries, with many countries employing clinical case definitions that could miss many cases (i.e., that were specific but not sensitive). WHO encourages countries to comply with the congenital syphilis surveillance case definition provided in the EMTCT guidance [11], which includes live births and stillbirths occurring among women with untreated or inadequately treated syphilis.

### Data verification and surveillance

Tracking of mother–infant pairs is critical to ascertain outcomes of all HIV- and syphilis-exposed children. Development of surveillance systems to document and track ANC, maternal and infant testing, and treatment require a sizeable informatics infrastructure. Infant tracking systems ensure appropriate referrals, testing, and treatment for HIV and syphilis after delivery. These were not well developed for syphilis-exposed infants in most countries. In general, support for HIV surveillance system development has exceeded that of syphilis, and this was evident when EMTCT criteria for syphilis were evaluated. Many countries do not track syphilis diagnosis in pregnant women, congenital syphilis cases, or stillbirths attributable to this infection. These limited syphilis surveillance systems challenge the calculation of congenital syphilis rates, a required indicator for EMTCT validation (≤50 cases/100,000 live births). Additionally, where the annual number of HIV-infected pregnant women is very low, a single transmission may result in a high MTCT rate, a situation experienced by some of the countries. This occurrence is taken into consideration by the global validation committee [11].

### Laboratory services

Quality assurance for HIV and syphilis testing is essential to EMTCT programming. Similarly, adherence to WHO-recommended testing algorithms ensures testing strategies are supported by performance evaluation and validation studies [9,22]. Internal and external quality assurance is needed for both HIV and syphilis testing. Laboratory quality assurance for syphilis testing was found to lag behind that of HIV in several countries, while others also lacked adequate quality assurance for HIV testing. Efforts to improve congenital syphilis surveillance and HIV and syphilis laboratory quality assurance should be included as key targets for country-based EMTCT programs.

### Human rights

WHO requires that HIV and syphilis EMTCT processes and indicators are achieved within a context of human rights, gender equality, and community engagement [20]. Interviews with human rights stakeholders as well as HIV-infected women are built into the country validation tools to identify human rights concerns. Each country validation is evaluated within the context of recent human rights violations, and countries are not held back from validation for activities that took place in the distant past. However, countries with recent or ongoing human rights violations related to EMTCT may not be able to receive validation despite achieving the process and target indicators. Many countries retain historical legislation criminalizing the transmission of STIs, including HIV. Still others may have recorded incidents of forced sterilization or forced abortion. In some countries, pregnant women less than 18 or 21 years of age cannot access HIV/STI testing and treatment services without parental consent. Violation of confidentiality among women with HIV is an ongoing concern, instances of which have been identified during the validation process.

### Special populations

Migrant and marginalized populations such as injecting drug users and sex workers can be a challenge for antenatal service delivery in countries on many different levels. Provision of universal equitable ANC services to citizen or noncitizen populations can present with resource considerations. Still further, documentation errors, treatment noncompliance, and limited medical follow-up of mobile populations may result in MTCT of HIV or syphilis despite best practices. Among the validated countries, some were challenged by undocumented internally displaced people, external migrants, refugees, or ethnic minority groups, some of whom were highly mobile and lacking any form of government identification. These populations may not access healthcare services and were perceived to be a potential reservoir of MTCT cases. Each of the validated countries provided free antenatal (inclusive of HIV and syphilis screening and treatment) and outreach services to these populations. Challenges related to special populations are taken into consideration during validation missions and case reviews to ensure that the validation process and tools are applied in a manner that is consistent with criteria in the guidance. MTCT events that occur despite standard screening and treatment practices are considered on an individual case by case basis in these situations during country program review by validation teams, and would not prevent a country from achieving validation [11].

## Discussion: Summary and way forward

Dual EMTCT of syphilis and HIV has been identified as a global public health priority and a priority in the context of the rights of a child to be born free of HIV and syphilis [23]. Criteria for validation of EMTCT include high levels of ANC access and HIV and syphilis testing and treatment for pregnant women and their infants. EMTCT priorities require integrated and universal access to these services within ANC and monitoring of coverage and health outcomes. The sentinel health system accomplishment of EMTCT validation demonstrates the political will by countries to improve the quality of, and access to, ANC and to reduce maternal and infant morbidity and mortality. While recognition of the efforts of the 5 countries described here is due, renewed support is needed for initiatives to achieve validation targets in many countries with high prevalence of HIV and syphilis, particularly those in sub-Saharan Africa.

Countries with high and low maternal HIV and syphilis prevalence face different challenges related to achieving the impact and process indicator targets of EMTCT. Countries with low-level prevalence may not have universal HIV and syphilis testing polices for pregnant women, thus making them ineligible to apply for validation. Countries with low-level but concentrated HIV or syphilis may have difficulty offering EMTCT services among hard-to-reach and high-risk populations. Countries with high burdens of HIV and syphilis encounter challenges, particularly with achieving the HIV case rate indicators because of ongoing MTCT despite 95% ANC, antenatal testing, and antenatal treatment coverage. These countries may reach elimination targets, albeit in the distant future, provided they can reduce maternal seroprevalence by reducing HIV incidence among women of child-bearing age and maximize ART coverage and viral suppression among HIV-infected women prior to, or early in, pregnancy. Regardless of prevalence, countries working to achieve EMTCT targets require continuous political commitment, strong integrated health systems, national technical expertise, and strong sustainable national capacities. Additional elements essential to EMTCT planning include collaboration of health and community settings involved in the fields of HIV and syphilis, including health policy formulation, integrated program planning, clinical implementation, and monitoring and evaluation.

EMTCT validation processes will continue to evolve and benefit from country validation experience. The findings from these few countries should not be interpreted as representative for those seeking EMTCT validation. Following the validation of the 5 countries described here, revisions to the global EMTCT guidance document proposed by country EMTCT programs, regional validation teams, and the global validation committee will be incorporated. First, the process indicator of maternal HIV treatment coverage will be increased from 90% to ≥95%. Second, countries will be provided with guidance for several aspects of syphilis surveillance that include defining, reporting, and tracking cases of congenital syphilis and stillbirths associated with maternal syphilis. Third, the need for laboratory quality assurance of HIV and syphilis testing will be highlighted to ensure that countries achieve high levels of testing performance. Fourth, the revised guidance will define intervals of HIV-exposed infant follow up based on maternal treatment and breastfeeding. Fifth, the revised global EMTCT guidance document will include the updated tools for use by country and regional validation teams to evaluate EMTCT programs both prior to and during country missions. Finally, criteria for recognition of countries with high HIV and syphilis prevalence that have made tremendous progress in prevention of MTCT will be included with this revision.

There are several innovative approaches to advance EMTCT programming. In 2015, the first rapid dual HIV–syphilis point of care test received WHO prequalification [24]. Several countries are evaluating these tests in ANC settings to improve dual screening for HIV and syphilis [25–27], and WHO has provided interim guidance on their use and interpretation [28]. A congenital syphilis estimation tool has been developed for use by countries with limited surveillance of congenital syphilis cases. This tool is intended to provide an estimate of congenital cases that can serve as an additional data verification point during validation planning and missions [29–31]. The Pan-American Health Organization (PAHO) and the WHO Western Pacific Region including China have expressed commitment to the “triple elimination” of MTCT of HIV, syphilis, and hepatitis B [16,32–34]. Guidance on this triple elimination is expected to be forthcoming in 2017 from both regions and will serve as the structure for expanding the global guidance to include EMTCT of hepatitis B in addition to HIV and syphilis [16,32]. Countries can enter and track progress on annual EMTCT indicators within the WHO Global AIDS Monitoring system [35]. These results can then be accessed through the WHO Global Health Observatory to monitor progress [36].

Efforts to achieve and maintain these targets require quality improvement and monitoring activities at multiple levels. Procurement of commodities such as HIV and syphilis diagnostic tests and medications is a critical component that is required to maintain EMTCT services. In particular, there is an urgent need to ensure stable supplies of benzathine penicillin for treatment of maternal and congenital syphilis [37]. Support for laboratory quality assurance is needed for syphilis and HIV to ensure that diagnostic tests are of appropriate quality and interpretation standards are met. Similarly, quality assurance is needed for point of care HIV and syphilis tests. Close follow-up of HIV and syphilis-exposed infants requires robust tracking systems. Finally, human rights issues must be included at all levels of program development. As countries continue to expand HIV and syphilis screening and treatment among pregnant women, fewer MTCTs will occur, but resources to maintain EMTCT programs are needed to prevent future resurgence.

WHO, along with multiple countries, is now embracing the commitment to reduce the MTCT of HIV and/or syphilis to elimination levels, such that these cease to be public health problems. This will require political advocacy and commitment from experts in HIV, syphilis, maternal child health, health policy, program implementation, and monitoring and evaluation. In order to eliminate MTCT of HIV and syphilis, countries will need to strengthen surveillance and laboratory systems, provide programmatic services to even the hardest to reach populations, and ensure that their program is based on core human rights, gender, and community engagement principles. Such efforts will contribute significantly not only to EMTCT of HIV and syphilis and better maternal and child health, but also to broader efforts to achieve universal access to healthcare and the SDGs.

## Supporting information

S1 Fig Timeline of EMTCT validation process for Thailand. (DOCX)

S2 Fig Three dimensions of UHC and EMTCT. (DOCX)

## Abbreviations

ANC: antenatal care
EMTCT: elimination of mother-to-child transmission
HBsAg: hepatitis B surface antigen
MTCT: mother-to-child transmission
PAHO: Pan-American Health organization
SDG: Sustainable Development Goal
STI: sexually transmitted infection
UHC: universal health coverage
UNAIDS: the Joint United Nations Programme on HIV/AIDS
UNFPA: United Nations Population Fund
