# Supplementary material for: Elimination of mother-to-child transmission of HIV and Syphilis (EMTCT): Process, progress, and program integration
Source: PLoS Med. 2017 Jun 27;14(6):e1002329. doi: 10.1371/journal.pmed.1002329 (PMC5486952; doi:10.1371/journal.pmed.1002329)
Supplement: S1 Fig — (DOCX) [file pmed.1002329.s001.docx]

**S1 Fig: Timeline of EMTCT Validation Process for Thailand**
