# Supplementary material for: Elimination of mother-to-child transmission of HIV and Syphilis (EMTCT): Process, progress, and program integration
Source: PLoS Med. 2017 Jun 27;14(6):e1002329. doi: 10.1371/journal.pmed.1002329 (PMC5486952; doi:10.1371/journal.pmed.1002329)
Supplement: S2 Fig — (DOCX) [file pmed.1002329.s002.docx]

**S2 Fig: Three dimensions of UHC and EMTCT**


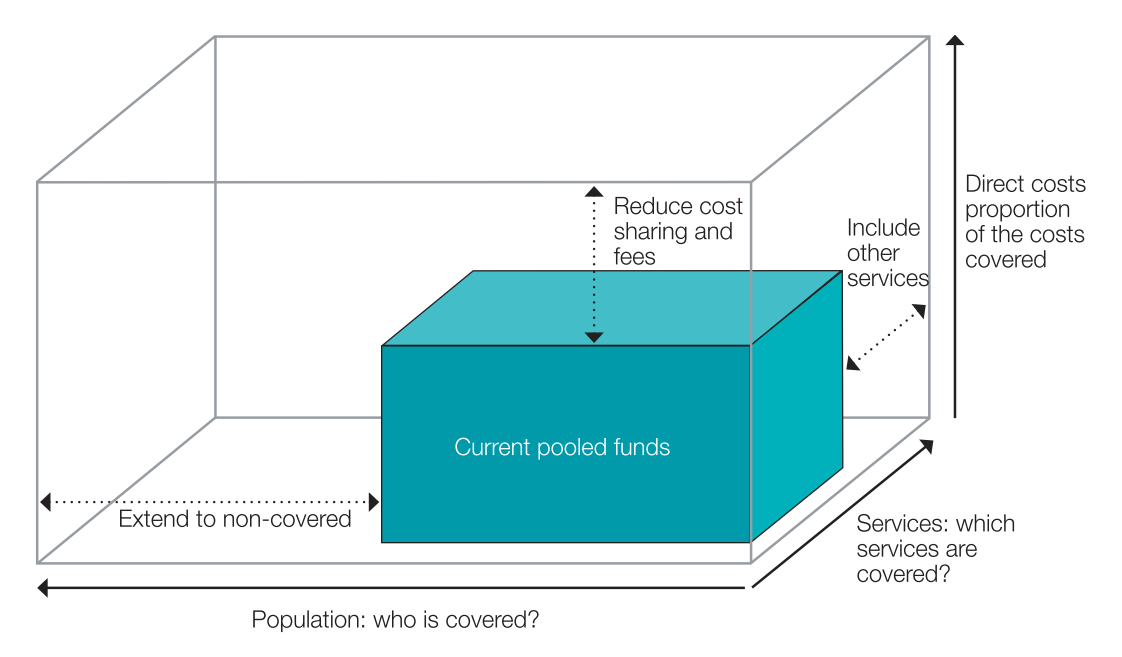


Are all PMTCT interventions included / covered?

Is quality of services ensured?

Are all pregnant women covered?

What proportion of PMTCT costs is covered?

Are human rights respected?

Is community engaged?
